# First Bite Syndrome: A Rare Post-Surgical Complication Following a Carotid Body Tumor Excision

**DOI:** 10.7759/cureus.76827

**Published:** 2025-01-02

**Authors:** Noura M AlOtaibi, Ali N Alanzan, Abdulaziz K Aloshaywi, Naif H Alotaibi, Abdulkarim F Alanazi, Faris M Alrajhi, Jawza Meaadi

**Affiliations:** 1 Oral and Maxillofacial Surgery, University of Glasgow, Glasgow, GBR; 2 Oral and Maxillofacial Surgery, King Saud University, Riyadh, SAU; 3 Dentistry, King Saud University, Riyadh, SAU; 4 Clinical Pharmacy and Neurology, King Saud Medical City, Riyadh, SAU

**Keywords:** first bite, maxillofacial tumor surgery, neck surgery, parotid pain, severe pain

## Abstract

First bite syndrome (FBS) is characterized by intense pain in the parotid area that starts with the first bite of food spontaneously. FBS is an uncommonly observed surgical complication of head and neck surgery. A 36-year-old male patient reported extreme pain after surgical excision of a carotid body tumor (CBT) in the ipsilateral parotid gland region at the first bite of each food intake, which improves gradually with continued mastication. Several factors are believed to be responsible for this phenomenon, including injury or amputation of the cervical sympathetic trunk. Loss of sympathetic innervation makes the parotid gland hypersensitive to parasympathetic stimulation. The best treatment for FBS is not yet firmly established, even though symptoms tend to improve with time in some cases. We report this case of FBS after CBT excision to raise awareness among maxillofacial surgeons of this surgical complication.

## Introduction

This report outlines an uncommon surgical complication following the surgical excision of a carotid body tumor (CBT), which is a first bite syndrome (FBS). The first description of FBS was published by Netterville et al. in 1998 [1]. FBS is a painful condition that can develop after the surgical excision of head and neck lesions, including CBT. The FBS symptoms are characterized by sharp pain associated with the first bite following a period of abstinence from eating. With subsequent bites, the pain gradually diminishes; therefore, the first bite and the first meal of the day are the most painful. Patients with FBS may tend to avoid eating due to the intensity of their pain [2].

It is believed that the pathophysiology of FBS remains to be elucidated, but it involves several interrelated mechanisms: nerve injury, particularly to the glossopharyngeal and vagus nerves, which disrupts normal sensory and motor function in the throat; neural plasticity, leading to altered sensory processing and heightened pain perception; sympathetic overactivity, which may amplify the sensory experience during mastication; and psychological factors, including anxiety and anticipation of pain, which can exacerbate symptoms [3]. Collectively, these factors contribute to the development of FBS, emphasizing the complex nature of its underlying mechanisms and the importance of targeted management strategies for patients with FBS [4].

Pathophysiology has been associated with an imbalance in the sympathetic and parasympathetic innervation of the parotid gland [3]. This is most frequently observed in the postoperative environment following surgery in the parotid or parapharyngeal space, although neoplastic causes have also been documented. Patients often exhibit simultaneous significant auricular neuropathy and/or Horner's syndrome [5]. Evidence on treatment is limited to case reports and series, but botulinum toxin injections and neuropathic medications have shown to be beneficial in certain cases.

The occurrence of FBS after excision of CBT is exceptionally rare, with only a few reports in the literature. This case study describes the case of a 36-year-old male who presented to the maxillofacial surgery clinic complaining of severe pain upon eating after CBT excision. It is important to increase the awareness of maxillofacial, head, and neck surgeons of this rare and underestimated complication.

## Case presentation

**Figure 1:**
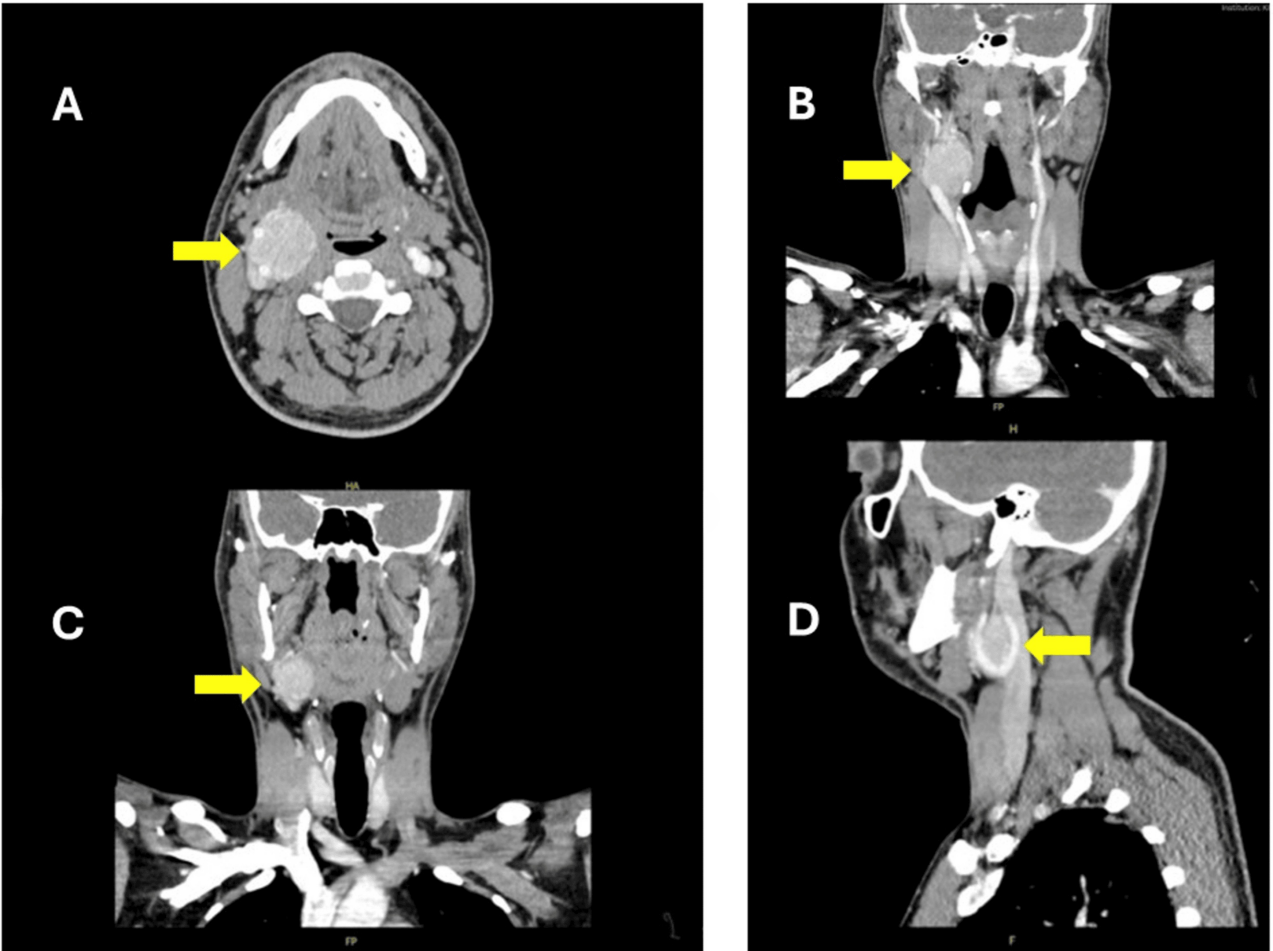
Perioperative computed tomography angiography scan of the neck (A) Axial view showing a well-defined ovoid mass measuring 3 x 4 cm located at the carotid bifurcation, (B) coronal view demonstrating the displacement of the internal and external carotid arteries laterally by the mass, (C) coronal view showing the anterior-medial extension of the mass, and (D) sagittal view illustrating the relation of the mass to adjacent parapharyngeal muscles

A 36-year-old male presented with right neck swelling of 16 months duration, accompanied by intermittent headaches. Imaging studies, including a neck ultrasound and CT scans, led to a diagnosis of a right CBT measuring 3 x 4 cm and adherent to the right external carotid artery (Figure 1). The patient underwent successful excision of the tumor with right external carotid artery ligation. Histopathology revealed a benign paraganglioma along with one reactive cervical lymph node. The postoperative course proceeded without complications; the patient initially encountered mild dysphagia and slight deviation of the tongue, which subsequently improved. Within a few days, he demonstrated the ability to tolerate a liquid diet, and his pain management was well-controlled. He was discharged on the fifth postoperative day without any significant complications.

However, during follow-up, the patient developed tenderness in the right facial region, including the temporomandibular joint (TMJ) area, along with paraesthesia extending from the midline of the chin to the ear. Despite these symptoms, his facial nerve function remained intact (House-Brackmann grade I), and there was no motor weakness. Notably, the patient reported ongoing discomfort in the right parotid area, which was aggravated by eating, particularly with the initial bite of food, a hallmark symptom of FBS. His symptoms are characterized by sharp, cramping pain in the parotid region triggered by the first bite of a meal, which then subsides with continued chewing. The pain starts at the right parotid area and radiates to the ear and eye of the ipsilateral side.

The patient’s ongoing discomfort and the timing of the symptoms strongly suggest that FBS after the exclusion of other possible causes of his symptoms. The FBS is a rare post-surgical complication and could have developed after his CBT excision. FBS is thought to arise from an imbalance in the sympathetic and parasympathetic innervation of the parotid gland, often following surgery in the parapharyngeal or parotid regions.

The patient was managed conservatively, with symptomatic relief provided through pain management and dietary adjustments. The botox option was given; however, the patient preferred to wait. Over time, his symptoms of FBS gradually improved, and he experienced full recovery within four months. There was no recurrence of the tumor, and the residual facial discomfort was completely resolved. At follow-up visits, the patient reported no further issues, and his facial nerve function remained normal. He was discharged with instructions for routine surveillance, which included a duplex ultrasound and ongoing monitoring for any potential late complications. His recovery was deemed complete, and he was advised to maintain regular follow-up care to ensure optimal long-term outcomes.

## Discussion

In this case study, we outlined the clinical course and management of a 36-year-old male patient who underwent surgical removal of a right CBT. The patient's postoperative recovery following the excision of the CBT was uneventful, displaying no signs of recurrence or complications throughout the follow-up period.

An interesting feature of this case was the development of FBS as a postoperative complication of CBTs, also called paragangliomas, which are uncommon neuroendocrine growths originating from the chemoreceptor cells in the carotid body. It should be noted that both conditions are extremely rare. The reduction of the patient's symptoms, along with the absence of any vascular abnormalities as evidenced by the duplex arterial examination following surgery, corresponds with the success of the surgical intervention for this rare tumor.

Regarding the patient's history of pain postoperatively, the differential diagnosis included TMJ dysfunction versus non-odontogenic pain origin. FBS is a rare pain disorder characterized by severe discomfort in the parotid area, provoked by the first bite of a meal, particularly when eating acidic foods [6]. The pathophysiology involves sympathetic denervation of the parotid gland, leading to parasympathetic hypersensitivity [7].

Although it is commonly seen as a complication following upper cervical surgery, FBS can also manifest as a primary symptom of malignancies in the parapharyngeal space [8,9]. The syndrome is commonly linked to sympathetic denervation and increased parasympathetic activity, often occurring alongside Horner's syndrome [6]. However, there have been reports of FBS occurring in the absence of Horner's syndrome, indicating that other pathogenic mechanisms may play a role [9]. Diagnosis of FBS commonly is based on the characteristic pain pattern, which diminishes with subsequent bites but recurs at the next meal [6].

FBS has been noted as a complication after surgeries for a range of tumors, such as paragangliomas, schwannomas, and adenoid cystic carcinomas [6,9]. The condition is difficult to treat, though the pain usually decreases with time [8,9]. When diagnosing FBS without a history of head and neck surgery, clinicians should consider examining the parapharyngeal space and deep parotid lobe [8,9].

FBS is difficult to manage with traditional analgesics. However, injections of botulinum toxin type A into the affected parotid have shown promise as an effective treatment by causing parasympathetic nerve impairment [7].

## Conclusions

This case report presents a unique instance of FBS as a late surgical complication of the excision of a CBT in a 36-year-old male. It highlights the importance of increasing awareness among maxillofacial surgeons and head and neck surgeons of FBS as a surgical complication post-surgical removal of CBTs. While the patient's initial recovery was uneventful, the development of facial pain related to FBS warrants further investigation and management. This case emphasizes the importance of comprehensive postoperative monitoring and timely intervention to address potential complications associated with CBT excision. Further studies are required to understand the underlying mechanisms of FBS, develop effective methods to avoid such complications, and provide treatment strategies for this challenging condition.
